# Evaluation of ivermectin antiviral activity against avian infectious bronchitis virus using a chicken embryo model

**DOI:** 10.1080/23144599.2022.2050077

**Published:** 2022-03-25

**Authors:** Donald L. Reynolds, E. Barry Simpson

**Affiliations:** School of Veterinary Medicine and Biomedical Sciences University of Nebraska – Lincoln, Lincoln, NE, USA

**Keywords:** Ivermectin, coronavirus, antiviral, infectious bronchitis virus

## Abstract

Ivermectin is widely used in both animals and humans as an FDA-approved parasiticide. Ivermectin has also been reported to have antiviral activity against several viruses including coronaviruses. There are reports that indicate ivermectin may have some role in diminishing the disease caused by SARS-CoV-2, but the evidence is inconclusive. The objective of this study was to determine if ivermectin was efficacious in inhibiting avian infectious bronchitis virus (IBV, a coronavirus) replication in chicken embryos. Briefly, our approach was to use the Massachusetts vaccine strain of IBV in combination with various doses of ivermectin and then inoculate these preparations into chicken embryos to determine if IBV replication was inhibited. The embryos were examined for IBV lesions and samples of chorioallantoic fluid were collected for IBV RT-PCR analysis. Several trials were performed, and the results of our study indicate that ivermectin did not inhibit IBV replication in chicken embryos.

## 1. Introduction

During the first quarter of 2020 the COVID-19 pandemic was spreading across the United States at an alarming rate with many afflicted people being hospitalized. The medical community developed extreme interest in searching for drugs to combat the disease. Many drugs received consideration. Although these drugs were FDA approved, they were approved for disease conditions and indications other than COVID-19. Some of these drugs received much attention in the popular press and included antiviral, anti-inflammatory and anti-parasitic drugs. Drugs that were evaluated included remdesivir, baricitinib, choloroquine/hydroxychloroquine, famotidine, ivermectin and others [1]. Ivermectin, which is widely used in the medical and veterinary professions as a parasiticide, received much attention after Dr. Pierre Kory testified at a U.S. Senate and Homeland Security and Governmental Affairs Committee on Capitol Hill, 8 December 2020, and stated: “ … that ivermectin is effectively a ‘miracle drug’ against COVID-19”. [2]. Subsequently, the National Institutes of Health issued guidelines on treating COVID-19 with ivermectin and stated: “There are insufficient data for the COVID-19 Treatment Guidelines Panel (the Panel) to recommend either for or against the use of ivermectin for the treatment of COVID-19. Results from adequately powered, well-designed, and well-conducted clinical trials are needed to provide more specific, evidence-based guidance on the role of ivermectin in the treatment of COVID-19”. [3]. Heidary and Gharebaghi published a systematic review that included the antiviral effects of ivermectin on various viruses [4]. This review reported on ivermectin antiviral effects conducted in *in vitro* models and included two avian viruses – Newcastle disease virus (NDV) and avian influenza virus. These reports piqued our interest in determining the potential of using ivermectin to combat coronaviral diseases of poultry. Therefore, the objective of this study was to determine if ivermectin had antiviral efficacy against the poultry coronavirus, avian infectious bronchitis virus, using a chicken embryo model.

## 2. Materials and methods

### 2.1. Ethical statement

This study was conducted at the University of Nebraska – Lincoln. The protocols were approved by the Institutional Animal Care and Use Committee (Project ID 2114).

### 2.2. Virus

The Massachusetts type of avian infectious bronchitis live vaccine virus (Merial, Inc., Athens, GA) was used. The same lot of vaccine virus was used for all trials. The lyophilized virus was supplied in vials containing 5,000 chick doses per vial. The lyophilized vaccine was first reconstituted with 10 mls sterile phosphate buffered saline (PBS) and then further diluted in PBS to a final dilution of 1:5000. The virus was then titrated in 10-day-old chicken embryos as follows (briefly): 10-fold serial dilutions of the vaccine virus were prepared using sterile PBS. Twelve chicken embryos per dilution were inoculated with 0.1 ml of diluted virus per embryo by the chorioallantoic (CA) route. Embryos were candled after 24 hours, and any dead embryos were removed. Following a 72-hour incubation period post-inoculation, approximately 0.5 ml of CA fluid was withdrawn from each egg and submitted for reverse transcriptase – polymerase chain reaction analysis (RT-PCR, see below) and the embryos were removed from their shells and examined for lesions. After determining which embryos were positive and negative by observing embryo lesions consistent with IBV infections and corroborating these results with RT-PCR, a median egg infectious dose (EID_50_) was determined by a previously published method [5]. The 1:5000 diluted vaccine was found to contain 80 EID_50_ per ml.

### 2.3. Eggs

Specific pathogen-free (SPF) eggs obtained from Valo BioMedia (Adel, IA) were used throughout the study. The fertile SPF eggs were delivered to our facility and were incubated in our laboratory to obtain the 10-day-old chicken embryos used in this study.

### 2.4. Virus detection

IBV infection of the embryos was determined by removing the embryos and observing characteristic IBV embryo lesions including stunting, curling, clubbing of the embryo down and urate deposits in the kidneys. These results were corroborated by testing the CA fluid by RT-PCR. RT-PCR BioChek (Scarborough, ME; https://www.biochek.com/poultry-pcr/ibv-pcr-infectious-bronchitis-virus-rna-test-kit/) kits were used, and the assays were performed in the Nebraska Veterinary Diagnostic Center as per the manufacturer’s instructions. A sample was considered positive with a cycle threshold (Ct) of 39 or less as per the manufacturer’s recommendation.

### 2.5. Ivermectin

Agri-mectin® Agri Laboratories, Ltd. (St. Joseph, MO) a 1% sterile solution was used. The molecular weight of ivermectin is 875 g/mol and was used for dose determination. The concentrations of ivermectin used for all trials were prepared in sterile PBS and are specified both in micromoles (uM) and in micrograms/millilitre (ug/ml). The ivermectin was combined with the IBV inoculum in separate sterile tubes prior to egg embryo inoculation at room temperature. These preparations were then injected into embryonated eggs at 0.1 ml per egg by the CA route within 30 minutes following preparation.

### 2.6. Experimental design, trial 1

Trial 1 contained eleven groups of chicken embryos that were inoculated at 10 days of incubation. Each group contained five chicken embryos except for group 11, which contained six. Groups 1 and 2 received no ivermectin and served as negative and positive virus controls, respectively. Groups 3 through 10 received ivermectin at various doses and were either inoculated with IBV and ivermectin or PBS (i.e. no virus) and ivermectin. The amount of ivermectin per chicken embryo for each group was as follows: groups 3 and 4 received 100 uM (87.5 ug/ml), groups 5 and 6 received 50 uM (43.75 ug/ml), groups 7 and 8 received 25 uM (21.9 ug/ml) and groups 9 and 10 received 12.5 uM (11 ug/ml). Group 11 served as an uninoculated control group. The IBV inoculum consisted of 80 EID_50_ per chicken embryo. At 72 hours post-inoculation embryos were removed from their eggs and grossly examined for signs of virus infectivity and/or ivermectin toxicity. Chorioallantoic fluid was also removed from each embryo and a pooled sample for each group of eggs was submitted for virus determination by RT-PCR analysis. The experimental design is also displayed in Table 1.

**Table 1.** Results of trial 1

Group	Ivermectin Dose uMug/ml		No. Embryos	Virus	RT-PCR (Ct)	No. Embryos with Lesions
1	0 (PBS)	0 (PBS)	5	No	-	None
2	0 (PBS)	0 (PBS)	5	Yes	+ (23.71)	5/5
3	100uM	87.5ug/ml	5	No	-	3/5*
4	100uM	87.5ug/ml	5	Yes	+ (18.46)	5/5
5	50uM	43.75ug/ml	5	No	-	None
6	50uM	43.75ug/ml	5	Yes	+ (14.16)	5/5
7	25uM	21.9ug/ml	5	No	-	None
8	25uM	21.9ug/ml	5	Yes	+ (12.78)	5/5
9	12.5uM	11ug/ml	5	No	-	No
10	12.5uM	11ug/ml	5	Yes	+ (14.97)	5/5
11	0	0	6	0	ND	None

ND = not done *lesions not consistent with IBV lesions

### 2.7. Experimental design, trials 2 and 3

Trials 2 and 3 were conducted as displayed in Table 2 using a virus inoculum of 80 EID_50_ per chicken embryo. Groups 1 and 2 were negative and positive virus control groups, respectively, and received no ivermectin. Groups 3 through 5 received virus inoculum that had been combined with various concentrations of ivermectin prior to inoculation. The dose of ivermectin for groups 3 through 5 were 50 uM (43.75 ug/ml), 25 uM (21.9 ug/ml) and 12.5 uM (11 ug/ml) respectively. The number of chicken embryos inoculated per group are displayed in Table 2. As in trial 1 above, the 10-day-old chicken embryos were harvested 72 hours post inoculation, removed from their shells, and examined for viral lesions. Chorioallantoic fluid was collected from each embryo and submitted for RT-PCR analysis.

**Table 2.** Results of trials 2 and 3

Group	Ivermectin Dose uMug/ml		No. Embryos	Virus	Embryos Positive by RT-PCR	No. Embryo with Lesions
Trial 2						
1	0 (PBS)	0 (PBS)	8	No	0/8	None
2	0 (PBS)	0 (PBS)	12	Yes	7/12	7/12
3	50uM	43.75ug/ml	12	Yes	10/12	10/12
4	25uM	21.9ug/ml	12	Yes	12/12	12/12
5	12.5uM	11ug/ml	12	Yes	12/12	12/12
Trial 3						
1	0 (PBS)	0 (PBS)	5	No	0/5	None
2	0 (PBS)	0 (PBS)	10	Yes	7/10	7/10
3	50uM	43.75ug/ml	12	Yes	10/12	10/12
4	25uM	21.9ug/ml	12	Yes	12/12	12/12
5	12.5uM	11ug/ml	12	Yes	12/12	12/12

### 2.8. Experimental design, trials 4 and 5

Trials 4 and 5 were conducted in the exact same way as trials 2 and 3 except the numbers of chicken embryos inoculated per group differed (see Table 3) and the virus inoculum was increased to 800 EID_50_ per egg embryo.

**Table 3.** Results of trials 4 and 5

Group	Ivermectin Dose uM ug/ml		No. Embryos	Virus	Embryos Positive by RT-PCR	No. Embryo with Lesions
Trial 4						
1	0 (PBS)	0 (PBS)	8	No	0/8	None
2	0 (PBS)	0 (PBS)	12	Yes	12/12	12/12
3	50uM	43.75ug/ml	12	Yes	12/12	12/12
4	25uM	21.9ug/ml	12	Yes	12/12	12/12
5	12.5uM	11ug/ml	12	Yes	12/12	12/12
Trial 5						
1	0 (PBS)	0 (PBS)	10	No	0/10	None
2	0 (PBS)	0 (PBS)	11	Yes	11/11	11/11
3	50uM	43.75ug/ml	11	Yes	11/11	11/11
4	25uM	21.9ug/ml	11	Yes	11/11	11/11
5	12.5uM	11ug/ml	11	Yes	11/11	11/11

## 3. Results

**Figure 1.**
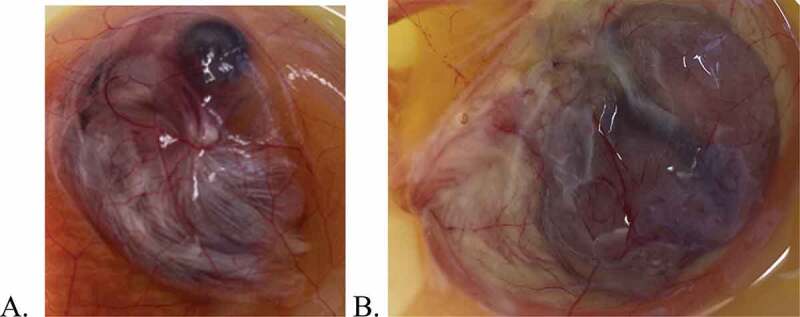
Chicken embryos at thirteen days embryonation. A. Uninoculated control embryo. B. Embryo inoculated at 10 days embryonation with 0.1 ml of 100 uM ivermectin.

The results of trial 1 are summarized in Table 1. No embryo lesions were observed nor were any RT-PCR results positive for any of the embryos that did not receive virus except for group 3. In group 3 (those embryos that received the highest dose of ivermectin (100 uM/87.5ug/ml) and no virus inoculum), three of five embryos displayed lesions but were not positive for IBV by RT-PCR. The three embryos appeared stunted and hyperaemic compared to controls (see Figure 1). Displayed in Figure 1A is an uninoculated control embryo at 13 days of embryonation. Figure 1B is the corresponding 13-day-old embryo that was inoculated with 100 uM of ivermectin (but no IBV) at 10 days of embryonation. The embryos are somewhat obscured by the presence of the CA membrane containing CA fluid; however, it can be observed that the embryo in 1B is stunted (i.e. smaller) compared to the embryo in 1A. The lesions displayed by these three embryos were not typical of lesions induced by IBV. The samples collected for RT-PCR analysis were pooled samples for each group and corroborated the results of embryo lesions due to virus infectivity (i.e. groups 2, 4, 6, 8 and 10). Results of trial 1 provided no indication of viral inhibition at any ivermectin dose.

The results of trials 2 through 5 are displayed in Tables 2 and 3. The embryo lesions observed in the virus-inoculated groups were corroborated with RT-PCR results. However, in groups 2 and 3, in trials 2 and 3, some embryos did not display the lesions typical of IBV infected embryos and the corresponding CA fluid samples were negative by RT-PCR. In groups 4 and 5 of trials 2 and 3, all embryos displayed lesions typical of IBV infection and all CA fluid samples were positive by RT-PCR. All embryos inoculated with IBV in trials 4 and 5 displayed typical IBV lesions and were positive by RT-PCR.

## 4. Discussion

This study was conducted using chicken embryos. Although some may consider this an *in vitro* study and recognizing that a chicken embryo is not a fully developed animal, we ascertain an embryo is more complex and emulates an *in vivo* model more so than *in vitro* models which employ cultured cells. Therefore, some may consider the results of studies using chicken embryos more relevant than *in vitro* results using cell culture. Also note that the use of chicken embryos was vetted by our institutional animal use and care committee.

Trial 1 was designed as a preliminary trial to determine the effect of the IB vaccine virus on 10-day-old chicken embryos and to evaluate the potential toxicity of ivermectin. That is, trial 1 was designed as a pilot trial to ensure our virus inoculum was infective and to evaluate any toxic effects that ivermectin may have had on chicken embryos in order for us to establish an ivermectin dose range and an infective IBV dose for the inoculum. Therefore, the experimental design included several groups with small numbers of chicken embryos in order to conserve resources and to minimize the use of chicken embryos while allowing us to explore an adequate dose range. We chose to use 80 EID_50_ as our IBV inoculum. This was based on the work of others using NDV in chicken embryo models to evaluate the antiviral effects of ivermectin and other substances in which 100 EID_50_ was used as the inoculum [6,7]. We realize that although NDV and IBV are both single-stranded RNA viruses, there are important differences between the two and an inoculum of 80 EID_50_ may not have been the optimum inoculum for our IBV chicken embryo model. Perhaps 80 EID_50_ was not sufficient to infect all embryos therefore, we increased the inoculum to 800 EID_50_ (please see below). Our initial dose range of ivermectin in trial 1 was determined by a previous published report by Azeem, et al. who reported cytotoxic effects of ivermectin using a chick primary embryo fibroblast cell line at concentrations greater than 50 ug/ml [6]. Based on our data from trial 1, and the report by Azeem et al., we attributed the lesions observed in the embryos from group 3 (ivermectin with no virus) to be toxic effects of ivermectin and we elected to eliminate the high dose (100 uM/87.5 ug/ml) from trials 2 through 5.

In trials 2 through 5 the numbers of chicken embryos per group varied in each trial due to the availability of chicken embryos at the time of the trial. In groups 2 and 3 of trials 2 and 3, not all virus inoculated chicken embryos displayed IBV lesions nor were they RT-PCR positive. Chicken embryos in group 2 of both trials 2 and 3 served as our positive IBV control groups. We expected all chicken embryos to be positive for IBV. However, as can be observed in Table 2, some chicken embryos were not infected by IBV as determined by the absence of embryo lesions and negative RT-PCR results. Additionally, some chicken embryos in group 3 for both trials 2 and 3 were also negative for IBV. If IBV negative chicken embryos had been found only in the high-dose ivermectin groups (group 3, trials 2 and 3), then we may have considered a viral inhibitory effect. However, since there were so few negative eggs in group 3 and realizing we had more negative eggs in group 2 (our positive viral control groups in trial 2 and 3) than group 3, we considered these results as experimental error. Such experimental error may have occurred in the inoculation procedure especially since we were using a relatively low dose of virus inoculum and placement of the inoculum may have varied due to the lack of technical experience on our part. Therefore, because a low number of chicken embryos were negative for IBV in groups 2 and 3, in trials 2 and 3, we repeated the trials increasing the viral inoculum by 10-fold (i.e. 800 EID_50_) in trials 4 and 5. As can be seen in Table 3 all virus inoculated chicken embryos were 100% positive for IBV as determined by embryo lesions and corroborated with RT-PCR results. One might argue that we merely “overloaded” the model by using a higher virus inoculum in trials 4 and 5. That is a valid argument and point well taken. However, since we observed no viral inhibition at the lower virus inoculum in trials 2 and 3, we are confident in stating that in the chicken embryo model we employed, ivermectin did not inhibit viral replication. Azeem et al. reported antiviral activity with ivermectin using a similar chicken embryo model and NDV. However, the ivermectin concentrations exhibiting antiviral activity were also considered cytotoxic [6].

Although SARS-CoV-2 and IBV are both coronaviruses we recognize there are important differences between the two. One report states that the genomes of the two viruses have only 43% identity [8]. SARS-CoV-2 is a betacoronavirus and utilizes the angiotensin converting enzyme 2 (ACE2) as its host cell receptor. In contrast, IBV is a gammacoronavirus and utilizes the sialic acid receptor [^9–11^]. SARS-CoV-2 does not infect chickens or chicken embryos [12,13] and IBV does not infect humans and has no known public health concerns [11]. Therefore, the use of IBV chicken embryo infection as a SARS-CoV-2 model system should be carefully evaluated. It has been reported that ivermectin has antiviral activity against SARS-CoV-2 in an *in vitro* cell culture system [14]. Antiviral activity of ivermectin has also been shown to occur in an *in vitro* cell culture system with an avian influenza virus [15]. There are other reports of antiviral activity of ivermectin against both RNA and DNA viruses [4]. The results of this trial do not support antiviral activity of ivermectin. However, under different experimental conditions and/or formulations, perhaps ivermectin could exert antiviral activity [16]. Perhaps adult chickens (or other animals) may be more tolerant to higher doses of ivermectin and allow antiviral activity to occur. It is recognized that there are various ways to design these types of trials. The rationale that was used in this study for combining the IBV and drug (i.e. ivermectin) and inoculating the chicken embryos was to ensure that the virus combined with the drug (i.e. ivermectin) were both deposited in the chicken embryo at precisely the same location and thus decreasing the risk of having the virus in one location (within the embryo) and the drug in another. This approach is frequently used in virus neutralization assays when testing antisera. Additionally, the rationale for combining the drug and virus inoculum and administering it to the chicken embryos within 30 minutes was to evaluate the inhibition of virus replication and not virus inactivation. Had we found indications of antiviral effects in our embryo model we would pursue further studies in an attempt to find applications for ivermectin to be used as an intervention/treatment strategy for coronavirus infections in poultry and other domestic animals. However, based on the results of this study we are less enthusiastic about pursuing further studies.

Negative research results are often deemed not worthy of reporting. However, because of the public health concerns and information being distributed about ivermectin, we believe the results of this study are germane. Unfortunately, there have been those that have consumed veterinary formulations of ivermectin in an apparent attempt to prevent or treat COVID-19 resulting in hospitalization [17].

## Conclusion

Therefore, we are compelled to report that we have no laboratory results that support antiviral activity of ivermectin against the Massachusetts vaccine strain of IBV that is used to prevent avian infectious bronchitis.
